# Identification of a novel SALL4 variant associated with unilateral renal agenesis and right renal pelvis duplication by prenatal exome sequencing: a case report

**DOI:** 10.3389/fped.2025.1535435

**Published:** 2025-07-30

**Authors:** Tingting Zhao, Jie Liu

**Affiliations:** Biochip Laboratory, Yantai Yuhuangding Hospital Affiliated to Qingdao University, Yantai, Shandong, China

**Keywords:** SALL4, unilateral renal agenesis, duplication of renal pelvis, WES, children

## Abstract

Congenital renal anomalies are one of the leading causes of perinatal and neonatal mortality in children. Here, we present a case of a 17-week-6-day pregnant patient, in whom prenatal ultrasound confirmed the fetal right duplex kidney and left renal agenesis, leading to termination of pregnancy at the patient's request. Whole-exome sequencing were conducted on the fetus and its parents to identify the cause of the fetal ultrasound abnormalities, followed by validation with Sanger sequencing and CMA/SNP-Array. Bioinformatics analysis assessed the pathogenicity of the mutation site using SIFT, PolyPhen-2, and Mutation Taster. A *de novo* heterozygous mutation c.486_487del(p.P163Hfs*17) was identified in exon 2 of the SALL4 gene, with neither parent carrying the mutation. The bioinformatics analysis results all support that the mutation is pathogenic. This frameshift mutation results in a complete alteration of the base sequence from the mutation site, leading to an abnormal amino acid translation and subsequent manifestation of the disease phenotype. Additionally, a maternally inherited 698.8 Kb deletion in the 2q13 region (seq[GRCh37] 2q13(110697011_111395836)x1) was detected in the fetus. Our study identified a novel *de novo* frameshift mutation in exon 2 of the SALL4 gene. This mutation is associated with unilateral renal agenesis and duplication of renal pelvis, providing valuable insights for genetic counseling and prenatal diagnosis of SALL4-related disorders.

## Introduction

Congenital anomalies of the kidney and urinary tract (CAKUT) are a major cause of perinatal and neonatal mortality in children, with renal agenesis accounting for over 20% of cases (1). Prenatally detected renal anomalies can be screened using various methods including imaging studies, biomarkers, family history, and genetic research, typically identified during abnormal fetal ultrasound scans between 18 and 21 weeks of gestation (2). Genetic variations play a certain role in the pathogenesis of CAKUT, with commonly implicated pathogenic genes including PAX2, EYA1, and HNF1B. However, the proportion of CAKUT patients who can be definitively diagnosed through genetic testing is currently around 10.3%, indicating that only a subset of cases can be explained by known genetic factors (3). PAX2 encodes a transcription factor that organized caudal descent of the nephric duct, emergence of the ureteric bud, branching morphogenesis (4). EYA1 deficiency results in an absence of ureteric bud outgrowth and a subsequent failure of metanephric mesenchyme induction (5). Despite the multitude of known pathogenic CAKUT genes, they are still insufficient to elucidate the etiology in all patients (6).

SALL4 (Spalt Like Transcription Factor 4) is a protein-coding gene located in the chromosomal region 20q13.13-13.2, comprising 4 exons and 8 zinc finger motifs (7). SALL4 plays a critical role in transcriptional regulation for stem cell maintenance and self-renewal, and is widely expressed in early embryonic development and tumors, with reduced expression in adulthood limited to the testes and ovaries (8, 9). Consequently, SALL4 has been recognized as a potential diagnostic marker and therapeutic target for various cancers, with its expression closely associated with malignant progression in gastric, endometrial, and testicular cancers (10–12). Studies have indicated that SALL4 regulates genes involved in presomitic mesoderm differentiation, neural genes within the mesoderm and somite formation (13). Mutations and functional deficiencies in SALL4 often lead to multiple organ defects, including the nervous system, limbs, kidneys, heart, and anorectal region, giving rise to several autosomal dominant hereditary disorders such as Duane radial ray syndrome, Okihiro syndrome, acro-renal-ocular syndrome, and IVIC syndrome (14).

Here, we investigated a 25-year-old pregnant woman, whose fetus was diagnosed with right duplex kidney and left renal agenesis at 17 weeks of gestation through ultrasound examination, and sought to identify the genetic cause of the fetal congenital renal anomalies through genetic sequencing.

## Case report

### Patient information

This case report describes a 25-year-old woman who, at 17 weeks and 6 days of gestation, was found to have a fetus with renal dysplasia detected by prenatal ultrasound. The patient has a history of good health, with no known chronic diseases, and denies any family history of hereditary diseases. Subsequent whole exome sequencing (WES) identified a novel, unprecedented mutation in the SALL4 gene. Consequently, we conducted a study involving the patient and her family members. Informed consent was obtained from the patient, and the study was approved by the Institutional Review Board.

**Figure 1:**
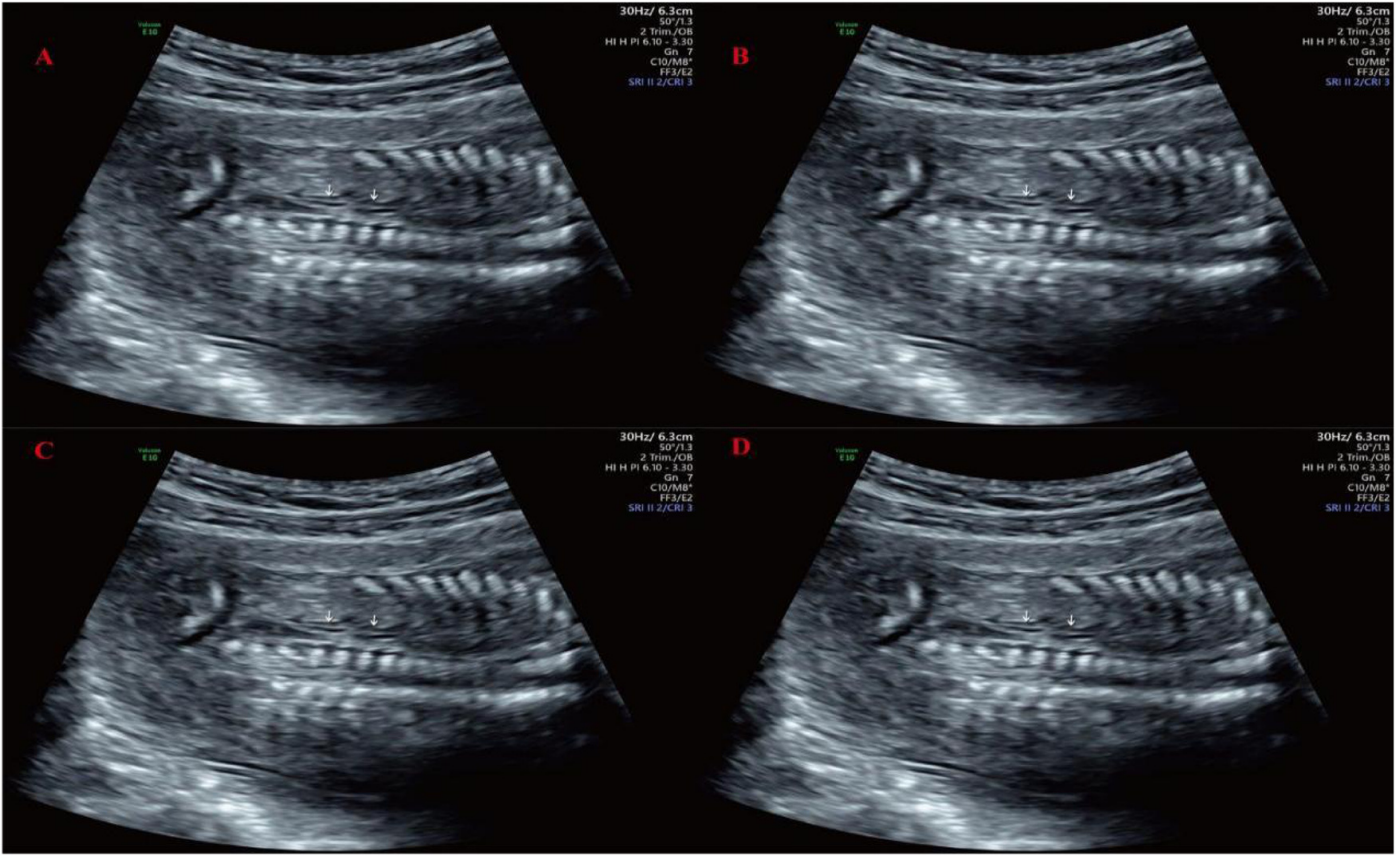
Prenatal ultrasound imaging at 17-week-6-day of gestation. **(A)** Adrenal gland lying-down sign; **(B)** Transverse section of the right kidney; **(C)** Coronal section of the right kidney; **(D)** Only the right renal artery was observed on Doppler imaging, with no identifiable course of the left renal artery.

The patient took dydrogesterone tablets during early pregnancy due to a history of threatened miscarriage, with a dosage of 100 mg twice daily for one week. At 17 weeks and 6 days of pregnancy, the prenatal ultrasound revealed the right kidney measuring approximately 2.4 × 1.2 cm, showing echoes of two renal pelvises with good echogenicity, and no apparent dilation of the renal pelvis or calyces, with no apparent abnormalities in the internal kidney structure. The left kidney region did not show distinct kidney structures, and the adrenal gland exhibited a “supine” sign (as shown in Figure 1). The maximum depth of the amniotic fluid dark area was 4.9 cm, with regular heartbeat and no other anomalies observed. The diagnosis suggested duplicated right kidney of the fetus and absence of the left kidney. Confirmation was made through a follow-up ultrasound examination three weeks later. The patient had no abdominal pain, vaginal bleeding, or discharge, and requested termination of the pregnancy. Given the presence of definite and severe renal developmental abnormalities in the fetus, medical termination of pregnancy was recommended in accordance with Article 18 of the Law of the People's Republic of China on Maternal and Infant Health Care. With full informed consent, the decision was approved by the pregnant woman and her family. The termination met the criteria for medical indication and was conducted in compliance with legal and ethical standards. At 23 weeks of gestation, an intra-amniotic injection of rivanol induction of labor was performed, resulting in the delivery of a stillborn male infant with normal external development. Fourteen months later, the patient became pregnant again and delivered a healthy baby boy.

### Whole exome sequencing

DNA quality was assessed using agarose gel electrophoresis to ensure no degradation, and DNA purity was measured by Nanodrop (OD260/280 = 1.8–2.0). Library construction and capture were performed using the SureSelect Human All Exon v6 Kit (Agilent Technologies, Santa Clara, USA), and sequencing was carried out on the Illumina next-generation sequencing platform using a paired-end (150 bp × 2) sequencing strategy to complete WES of the samples. The average coverage depth of the fetal sample was 116.92X, with 96.80% of the targeted regions covered at a depth greater than 10X and 91.68% covered at a depth greater than 20X. The average coverage depth of the maternal sample was 139.79X, with 98.33% of the targeted regions covered at a depth greater than 10X, and 96.05% covered at a depth greater than 20X. The average coverage depth of the paternal sample was 117.67X, with 97.85% of the targeted regions covered at a depth greater than 10X, and 94.29% covered at a depth greater than 20X. Data analysis was conducted using the BWA software (v0.7.17) (15) for alignment based on the GRCh37 (hg19) genome version. Base quality recalibration was performed using the Base Recalibration module of the GATK software (v4.2.0.0) (16). The likely pathogenic variant was classified according to the American College of Medical Genetics and Genomics (ACMG) standards and guidelines for the interpretation of sequence variants (17).

### Sanger sequencing

Detection was based on the Sanger sequencing platform. The extracted DNA was amplified by PCR and detected by ABI 3130XLsequencing platform. Data analysis was performed with reference to the GRCh37 (hg19) genome version.

### CMA/SNP-array

DNA was hybridized with the Affymetrix CytoScan™ 750K SNP-Array (Affymetrix, Santa Clara, CA, USA). After washing with the Affymetrix GeneChip Fluidics Station 450, the array was scanned using the GeneChip System (GCS) 3000Dx. Data analysis was performed with reference to the human genome GRCh37 (hg19).

### Pathogenicity analysis

SIFT (http://sift.jcvi.org/) (18), PolyPhen-2 (http://genetics.bwh.harvard.edu/pph/) (19), Mutation tater (https://www.mutationtaster.org/) (20) were used to assess the pathogenicity of the SALL4 gene mutation site.

### Gene testing results

**Figure 2:**
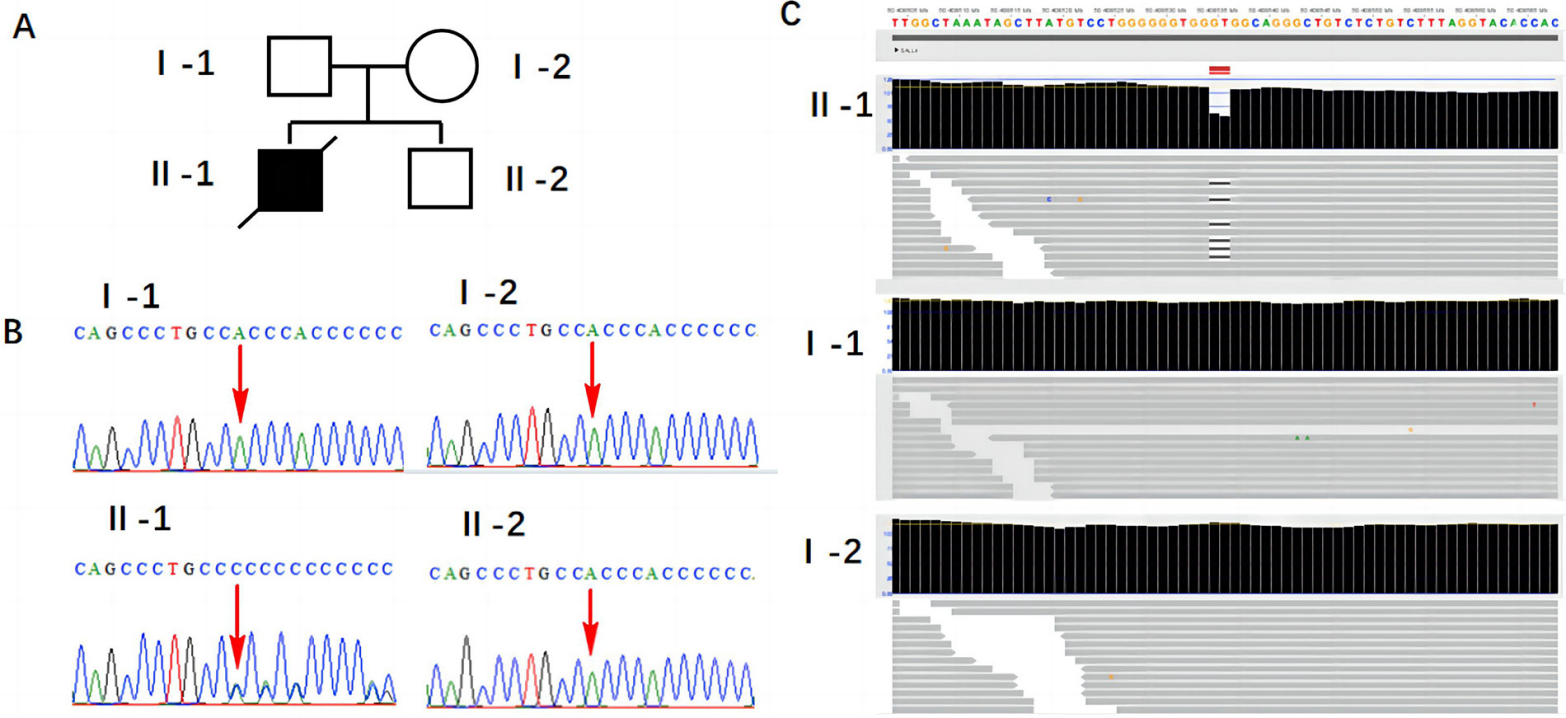
Results of SALL4 gene mutation detection. **(A)** Family pedigree: I-1: the patient's husband; I-2: the patient; II-1: the first child; II-2: the second child. **(B)** Sanger sequencing validation results. **(C)** WES detection results.

**Figure 3:**
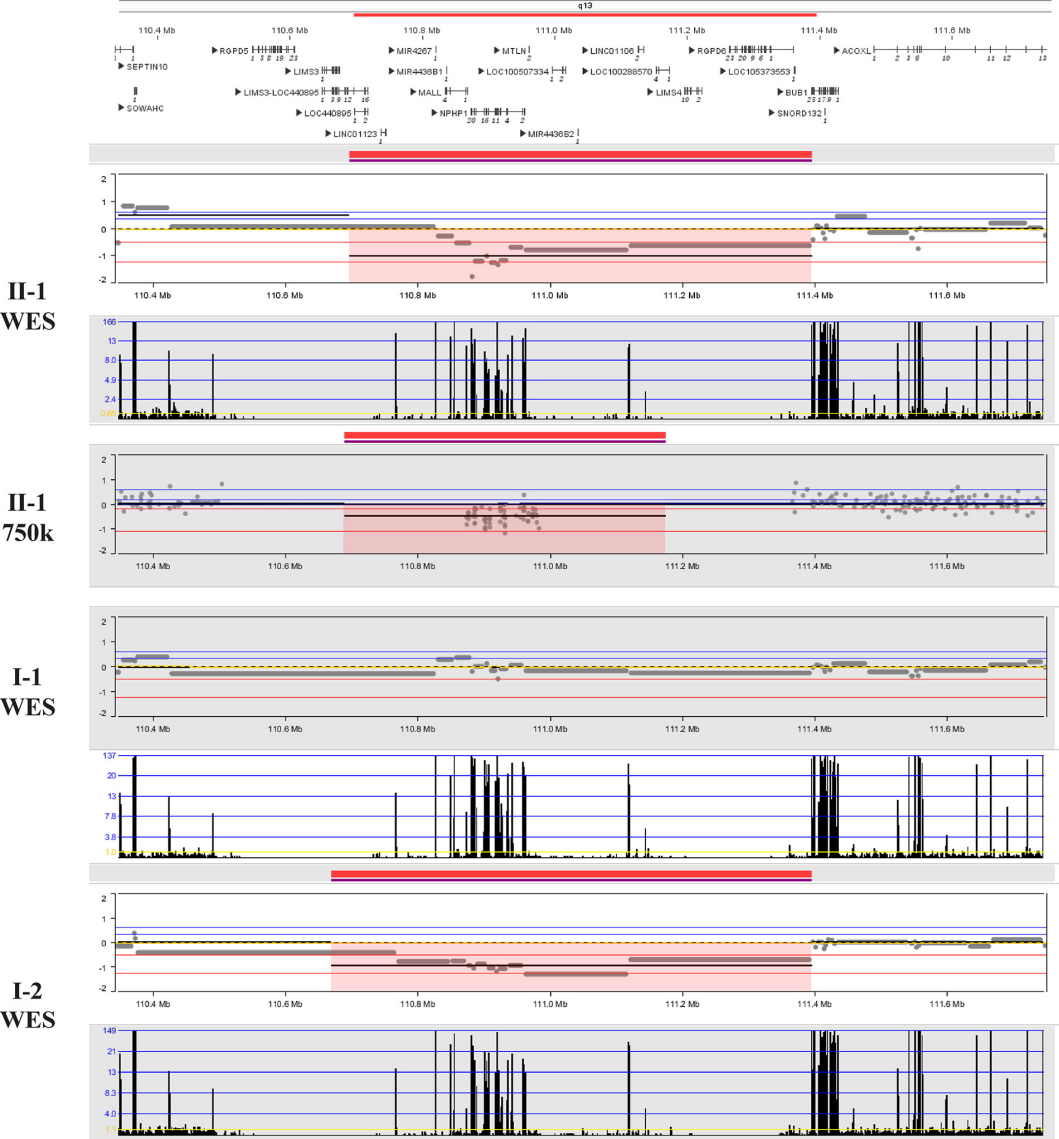
2q13 deletion on chromosome.

After obtaining informed consent from the patient, peripheral blood samples from the patient and her husband, fetal tissue samples, and amniotic fluid from the second fetus were collected. Genomic DNA was extracted from all samples using the MagMAX™ DNA Multi-Sample Ultra 2.0 Kit (USA), following the manufacturer's instructions. The extracted DNA was subsequently subjected to whole-exome sequencing (WES) and Sanger sequencing. The results revealed a *de novo* heterozygous frameshift mutation c.486_487del CC (p.P163Hfs*17) in exon 2 of the fetal SALL4 gene, corresponding to the genomic position chr20:50408535-50408536 (GRCh37), as shown in Figure 2. This mutation results from a deletion between the 486th and 487th base pairs of the gene, leading to the substitution of proline (Proline, P) with histidine (Histidine, H) at position 163 of the corresponding protein. Subsequently, a frameshift mutation occurs, introducing a premature stop codon at position 17. This implies that translation is terminated prematurely, potentially producing a truncated, non-functional protein. However, this mutation was not detected in the genes of the parents and the second child. This frameshift mutation causes a complete alteration of the base sequence from the point of damage, resulting in the translation of abnormal amino acids and leading to the corresponding disease phenotype. Additionally, we identified a 698.8 kb deletion in the 2q13 segment of the fetus inherited from the mother (seq[GRCh37] 2q13(110697011_111395836)x1), consistent with the Affymetrix CytoScan 750K SNP-Array results (arr[hg19] 2q13(110873835_110980295)x1). As shown in Figure 3, this region contains the NPHP1 gene.

The pathogenicity prediction results for the SALL4 gene variant, c.486_487del (p.P163Hfs*17), are summarized in Table 1. The SIFT algorithm classified the variant as “Damaging” with a score of 0, suggesting a detrimental impact on protein function. PolyPhen-2 predicted it to be “Possibly damaging” with a high score of 0.938, also indicating a high risk of pathogenicity. Mutation Taster directly classified the variant as “Disease causing”, consistent with this frameshift mutation resulting in a truncated protein. The consistent predictions from these three tools, based on different algorithms, provide strong support for the pathogenic nature of the SALL4 variant.

**Table 1:** SALL4 gene c.486_487del(p.P163Hfs*17) was predicted by SIFT, polyPhen-2, and mutation taster software.

Analysis tool	Gene	Risk prediction	Score
SIFT	SALL4 c.486_487del (p.P163Hfs*17)	Damaging	0
PolyPhen-2		Possibly damaging	0.938
Mutation taster		Disease causing	PhyloP: −0.092
			PhastCons: 0

PhyloP (Phylogenetic *P*-values): indicates site-specific conservation across species; PhastCons: measures the probability of a nucleotide being part of a conserved element.

## Discussion

This case report describes an 18-week pregnant patient, in whom ultrasound examination revealed unilateral renal agenesis and duplication of renal pelvis in the fetus. Subsequent genetic testing identified a novel *de novo* heterozygous mutation c.486_487del (p.P163Hfs17) in exon 2 of the fetal SALL4 gene, which is a novel one that has been discovered so far, along with a 698.8 kb deletion in the 2q13 segment inherited from the mother (seq[GRCh37] 2q13(110697011_111395836)x1). This finding provides important clues for elucidating the role of genetic variation in the mechanisms underlying renal developmental abnormalities.

Although the deletion of the segment 2q13(110697011_111395836) has not been specifically reported, and its clinical significance is not fully established, most studies suggest that such deletions are more associated with developmental delay and mild facial dysmorphia (https://doi.org/10.1002/ccr3.4289). For example, Eva et al. reported two patients with chromosome 2q13 deletions: Patient 1 (chr2:111415137-113194067 bp) presented with developmental delay, microcephaly, and mild dysmorphic facial features; Patient 2 (chr2:110980342-113007823 bp) exhibited autism spectrum disorder, borderline cognitive abilities, attention and executive function deficits, and mild dysmorphic facial features (21). This region contains the NPHP1 gene. Mutations/deletions of this gene are associated with the autosomal recessive juvenile kidney wasting disease (Nephronophthisis 1, juvenile), in which pathogenic variants account for approximately 20%–25% of the total cause of morbidity (22). Several studies have reported homozygous deletion of NPHP1 gene resulting in chronic renal failure (23, 24). In this case, both the fetus and the mother were heterozygous carriers of the NPHP1 deletion, which is consistent with an autosomal recessive inheritance pattern and is typically insufficient on its own to cause classical NPHP1-related nephropathy. However, the 2q13 deletion region may also encompass other developmentally relevant genes or regulatory elements, and haploinsufficiency of these loci may exert modifying or synergistic effects in the context of the *de novo* SALL4 variant. Therefore, although current evidence supports the SALL4 variant as the primary pathogenic factor, we cannot exclude the possibility that the 2q13 deletion may have a synergistic or additive impact on the phenotype.

It is noteworthy that both parents of the fetus were phenotypically normal, with no congenital renal abnormalities, and genetic testing did not detect any SALL4 mutations in either parent. Thus, the heterozygous frameshift variant c.486_487del (p.P163Hfs*17) in exon 2 of the SALL4 gene represents a *de novo* mutation rather than an inherited one. As such, the risk of recurrence in future offspring is extremely low. Follow-up after delivery showed that the couple subsequently gave birth to a healthy male infant with no clinical abnormalities the following year. This outcome provides a practical reference for genetic counseling in similar cases involving comparable genetic variants.

Sall4-related diseases are inherited in an autosomal dominant manner. The proportion of cases caused by new pathogenic variants is about 40%–50% (25). The autosomal dominant genetic diseases associated with SALL4 gene mutations include the IVIC syndrome (IVIC, OMIM:147750), characterized by upper limb abnormalities (radial ray defects, wrist bone fusion), dysmotility of extraocular muscles, and congenital bilateral non-progressive mixed hearing loss (26). Duane-radial ray syndrome (DRRS, OMIM:607323), also known as Okihiro syndrome, is mainly characterized by unilateral or bilateral Duane anomalies and radial ray malformations. The acro-renal-ocular syndrome (AROS) is characterized by radial ray malformations, renal abnormalities, and ocular tissue defects. In rare cases, pathogenic variations in the SALL4 gene can also lead to the typical Holt-Oram syndrome (HOS), with radial ray malformations and heart malformations without other features (25). Currently, more than fifty mutations related to SALL4 have been identified, with most of these mutations concentrated in exons 2 and 3 (27).

Mobarakeh reported a case of DRR syndrome in an Iranian patient, presenting with kyphoscoliosis, anterior sacral meningocele, barrel-shaped chest, and aortic disc. A novel *de novo* heterozygous nonsense mutation (c.712 C>T: p.Q238X) was identified in exon 2 of the SALL4 gene (9). Recently, researchers also identified, for the first time, a patient with DRR syndrome carrying a pathogenic heterozygous c.3060delG mutation in exon 4 of the SALL4 gene, characterized by skeletal abnormalities in the arms and hands and sensorineural hearing loss (8). SALL4 is closely related to kidney development, and previous case reports have shown that SALL4 mutations can cause renal developmental disorders. For example, patients with heterozygous 128 kb deletion of SALL4 manifested renal hypoplasia, radial ray and atrial septal defects, and patent ductus arteriosus (28). In a proband with bilateral asymmetrical radial ray anomalies and pelvic kidney developmental disorders, a heterozygous variant c.1717C>T in the SALL4 gene was identified. His father and grandfather, who had the same variant, exhibited bilateral asymmetrical radial ray anomalies and pelvic dystopia of the kidney (29). In this case, prenatal ultrasound findings showed that the fetal limb lengths and thumb structures were within normal ranges, with no abnormalities observed in the forearms, facial features, or ocular motility. Apart from the evident renal developmental anomalies, no structural abnormalities were detected in other organ systems. The available evidence is insufficient to support a diagnosis of DRR syndrome. Therefore, we consider the c.486_487del mutation in the SALL4 gene to be the most likely cause of the fetal renal developmental abnormality in this case. Although many studies have demonstrated the association between SALL4 mutations and renal dysplasia, the specific effects and mechanisms of SALL4 mutations on the kidneys have not yet been fully elucidated.

This report presents a novel SALL4 mutation leading to right-sided duplication of renal pelvis and left-sided renal aplasia, enhancing our understanding of the impact of SALL4 mutations on renal development. However, this study has certain limitations. Due to constraints related to the angle, resolution, and clarity of the fetal ultrasound images, the number of ureters associated with the right-sided duplex kidney could not be clearly identified, making it difficult to determine whether it was a complete duplication. In addition, this study reports only a single case, and therefore the interpretation of the results should be approached with caution. Further research is necessary to elucidate the underlying pathogenic mechanisms. Considering the coexistence of a maternally inherited 2q13 deletion and the atypical phenotype of the fetus relative to classical SALL4-related syndromes, we believe that future studies should further investigate the individual contributions of these two genetic alterations as well as their potential interactions. Consequently, we recommend comprehensive genetic analyses and in-depth functional studies in cases presenting with similarly complex renal anomalies and multiple potential genetic variants, in order to more accurately determine their etiological basis.

In summary, this case study reports a newly identified frameshift mutation on exon 2 of the SALL4 gene, which represents a novel variant identified to date. Unlike the typical radial ray defects commonly associated with SALL4 mutations, the fetus with this novel mutation primarily exhibited unilateral renal agenesis and duplication of the renal pelvis. These types of renal dysplasia have not been previously reported in cases of SALL4 mutations. This discovery expands the mutational spectrum of SALL4, contributing to better genetic counseling and prenatal diagnosis for SALL4-related disorders.
